# A Two-Step Machine Learning Approach Integrating GNSS-Derived PWV for Improved Precipitation Forecasting

**DOI:** 10.3390/e27101034

**Published:** 2025-10-02

**Authors:** Laura Profetto, Andrea Antonini, Luca Fibbi, Alberto Ortolani, Giovanna Maria Dimitri

**Affiliations:** 1 Dipartimento Ingegneria dell’Informazione e Scienze Matematiche (DIISM), Universitá di Siena, 53100 Siena, Italy; 2 Laboratory of Monitoring and Environmental Modelling for the Sustainable Development (LaMMA), 50019 Sesto Fiorentino, Italy; 3 Institute for the Bioeconony (IBE), National Research Council (CNR), 50019 Sesto Fiorentino, Italy; 4 Dipartimento SPS, Universitá Degli Studi di Milano, Via Festa del Perdono 7, 20122 Milan, Italy

**Keywords:** GNSS meteorology, Precipitable Water Vapor (PWV), precipitation nowcasting, machine learning, short-term weather forecasting

## Abstract

Global Navigation Satellite System (GNSS) meteorology has emerged as a valuable tool for atmospheric monitoring, providing high-resolution, near-real-time data that can significantly improve precipitation nowcasting. This study aims to enhance short-term precipitation forecasting by integrating GNSS-derived Precipitable Water Vapor (PWV)—a key indicator of atmospheric moisture—with traditional meteorological observations. A novel two-step machine learning framework is proposed that combines a Random Forest (RF) model and a Long Short-Term Memory (LSTM) neural network. The RF model first estimates current precipitation based on PWV, surface weather parameters, and auxiliary atmospheric variables. Then, the LSTM network leverages temporal dependencies within the data to predict precipitation for the subsequent hour. This hybrid method capitalizes on the RF’s ability to model complex nonlinear relationships and the LSTM’s strength in handling time series data. The results demonstrate that the proposed approach improves forecasting accuracy, particularly during extreme weather events such as intense rainfall and thunderstorms, outperforming conventional models. By integrating GNSS meteorology with advanced machine learning techniques, this study offers a promising tool for meteorological services, early warning systems, and disaster risk management. The findings highlight the potential of GNSS-based nowcasting for real-time decision-making in weather-sensitive applications.

## 1. Introduction

In recent years, an alarming global rise in the frequency and severity of flood-related disasters, driven by climate change, is underscoring the critical need for improved forecasting, infrastructure resilience, and early warning systems. In Derna, Libya in 2023, Storm Daniel caused two dams to collapse, killing over 5900 people and displacing tens of thousands [1]. In Nigeria, severe floods in 2022 left over 600 dead and 1.4 million displaced, partly due to upstream dam releases [2]. Southern Brazil faced its worst flood in 80 years in 2024, with 181 deaths and USD 3.7 billion in damages [3]. Meanwhile, Europe saw widespread river flooding in 2023, affecting 1.6 million people and causing 81% of the continent’s climate-related economic losses [4]. In Valencia, Spain, 228 people died in the 2024 floods, prompting criminal investigations over emergency response failures [5].

These cases illustrate how climate change, inadequate infrastructure, and governance failures intersect to turn extreme weather into large-scale disasters. The intensification of rainfall, coupled with land use changes and urbanization, has made many regions more vulnerable than ever. They serve as a stark reminder of the urgent need to invest in predictive technologies, robust infrastructure, and international cooperation to reduce disaster risks in a warming world.

Against this backdrop, accurate precipitation forecasting emerges as a critical tool for anticipating extreme weather events and supporting timely emergency response. Yet it remains a key challenge in meteorology, with significant implications for hydrology, agriculture, and disaster risk management. Traditional numerical weather prediction (NWP) models, while powerful, often require substantial computational resources and can struggle with localized and short-term forecasts [6,7]. In recent years, Global Navigation Satellite System (GNSS) meteorology has emerged as a valuable source of atmospheric information, particularly through the estimation of Zenith Path Delay (ZPD), which can be decomposed into the Zenith Hydrostatic Delay (ZHD) and the Zenith Wet Delay (ZWD). The ZWD component is closely related to atmospheric water vapor and can be used to derive Precipitable Water Vapor (PWV), a key variable in understanding and predicting rainfall events [8,9].

Several studies have demonstrated the potential of GNSS-derived PWV in precipitation nowcasting and forecasting. PWV has been shown to correlate well with rainfall intensity and occurrence, particularly in convective systems [10,11]. When integrated with traditional surface meteorological measurements such as temperature, pressure, humidity, and wind, GNSS data provide a richer characterization of atmospheric conditions.

Simultaneously, the field of machine learning (ML) has offered new tools for improving forecasting performance. ML algorithms, including decision trees, random forests, support vector machines, and deep neural networks, are capable of capturing complex nonlinear relationships between input variables and precipitation outcomes [12,13]. Recent research has shown that ML models can outperform conventional statistical techniques and even some NWP models in short-term precipitation prediction, especially when trained on diverse and high-resolution datasets [14,15].

From an information-theoretic perspective, atmospheric prediction tasks such as precipitation nowcasting can also be viewed through the lens of entropy and uncertainty reduction. Entropy-based metrics provide a quantitative framework for evaluating the information content and relevance of input features, aiding feature selection and model interpretability. In particular, techniques such as mutual information and entropy minimization have been applied to improve model performance in meteorological applications [16,17].

A recent approach introduced a mutual information-based regularization mechanism into deep learning architectures for precipitation nowcasting using radar imagery [18]. By maximizing the mutual information between input radar sequences and predictive outputs, the method encourages the model to preserve informative spatiotemporal structures while suppressing redundant features. This leads to improved accuracy and generalization, particularly in fast-changing atmospheric scenarios. Although developed in the context of radar data, the methodology illustrates how information-theoretic constraints can enhance the predictive power of neural networks in meteorological forecasting.

Furthermore, neural networks trained under information bottleneck or minimum entropy criteria have shown enhanced generalization and robustness in noisy or high-dimensional settings [19,20]. This theoretical foundation complements empirical machine learning approaches, offering deeper insight into the flow of information from GNSS signals and meteorological measurements to predictive outputs.

This study explores the integration of GNSS-derived atmospheric delay parameters (ZPD, ZWD, PWV) with surface meteorological data using machine learning models to improve precipitation forecasting. By leveraging the strengths of both data-driven methods and GNSS-based sensing, we aim to enhance forecast accuracy and support the development of operational tools for early warning systems.

The methodology consists of an initial phase in which the Random Forest Regressor (RFR) model was employed to estimate precipitation intensity at a given moment. Thanks to its ability to analyze large datasets and identify complex correlations, this model effectively captures the importance of PWV in precipitation forecasting. The output of the RFR, specifically the instantaneous precipitation intensity, was then used as the input for a Long-Short Term Memory (LSTM) model. LSTM models, a specialized type of recurrent neural network, are particularly effective in processing sequential data and capturing temporal dependencies in meteorological events. By incorporating past observations, the LSTM model was able to enhance the overall accuracy of precipitation forecasts. The forecasting approach aimed to predict both the maximum precipitation intensity and the cumulative precipitation with lead times of one hour and two hours.The experiment demonstrated that a two-tiered approach where data first undergo a preprocessing phase for noise reduction and are subsequently processed by an LSTM model for prediction significantly improves system performance, particularly in the absence of cumulative rainfall as an input parameter. The integrated analysis of GNSS data, combined with the implementation of advanced monitoring systems based on PWV and machine learning techniques, can be a crucial step in strengthening territorial resilience. The proposed model serves as a support tool for continuous monitoring, even in emergency situations.

## 2. Materials and Methods

### 2.1. Dataset and Preprocessing

The analyzed dataset comprises two files containing parameters collected between 2021 and 2023 from the GNSS meteorology station located on the roof of the LaMMA Consortium (located in Sesto Fiorentino, Tuscany). One file includes surface weather station data, while the other contains information related to the estimation of ZPD.

In the first file, only the relevant meteorological parameters were selected, including atmospheric temperature (TMA), relative humidity (RHU), atmospheric pressure (PRA), cumulative rainfall (RNC), and rainfall intensity (RNI). It is important to distinguish between cumulative rainfall and rainfall intensity; the former represents the total amount of precipitation accumulated over a specific period at a given location, expressed in millimeters (mm), whereas the latter measures the rate at which rain falls, typically expressed in millimeters per hour (mm/h). Rainfall intensity measures are useful for assessing the severity of a rain event at a given moment, while cumulative rainfall data provides insight into the overall impact over time.

The second file contains data on Julian day, latitude (lat), longitude (lon), altitude (height), and ZPD. The latter was obtained by processing the RINEX observation files generated by the GNSS receiver with the MG-APP (Multi-GNSS Automatic Precise Positioning) software [21], using the settings summarized in Table 1 The two files were merged by temporally aligning their respective records, considering that the meteorological data were sampled every 5 s, while the ZPD data were available every 30 s (at the same resolution of the available clock corrections).

The dataset included some missing values; however, since they accounted for only 1.94% of the total records, they were removed during preprocessing.

ZPD estimates were also used to compute the PWV, defined as the total amount of water vapor contained in a vertical column of the atmosphere, expressed in kg m^−2^.

The following is a brief description of the steps taken for its computation. The model to estimate the ZHD from the measurement of surface pressure *P*[hPa], station latitude φ, and height *h*[m] is ([22])

(1) $$\mathrm{ZHD}=2.27672768\cdot \frac{P}{1-0.00266\cdot \cos(2\varphi )-0.00028\cdot h}$$

Then, ZWD is generated by subtracting ZHD from ZPD. The Precipitable Water (PWV), considering the density of water ρ

(2) $$PWV=10^{6}\frac{ZWD}{\rho R_{w}\left(K_{2}^{\prime }+\frac{K3}{T_{m}}\right)}$$

with $K_{2}^{\prime }=K_{2}-K_{1}\frac{R_{d}}{R_{w}}=K_{2}-K_{1}\epsilon$. $K_{2}^{\prime }=22\;K\;{\mathrm{hPa}}^{-1}$, $K_{3}=3.739\times 10^{5}\;K^{2}{\mathrm{hPa}}^{-1}$, $R_{w}=461.495\;J\;{\mathrm{kg}}^{-1}K^{-1}$, according to [23].

$T_{m}$ is the weighted mean temperature and can be estimated from the surface temperature $T_{s}$[K] through the following linear model

(3) $$T_{m}=0.72T+70.2,$$

Since the variables influencing ZPD and PWV (temperature, pressure, and water vapor) exhibit lower time variations, we chose to aggregate the data to a 10 min sampling interval. This aggregation was also selected to reduce high-frequency noise and improve the model’s generalization capability. The ZPD values produced from the MG-APP software processing showed a mean of 2425.5 mm, with a standard deviation of 51.5 mm, a minimum of 2282.0 mm, and a maximum of 2706.2 mm. These statistics provide an overview of the variability of the dataset.

Before implementing any predictive models, two essential preprocessing steps were applied to prepare the time series data for ingestion by a neural network.

The first step involved data normalization. Time series data typically include variables with differing magnitudes and units, which can adversely affect model training. To ensure that each feature contributed proportionally to the learning process, all variables were standardized to the range [−1,1] by subtracting the mean and dividing by the standard deviation of each series. It is important to note that the mean and standard deviation were computed exclusively on the training set and subsequently applied to both the validation and test sets.

The second step consisted of formatting the data into a suitable input structure for the model. Specifically, the dataset was transformed into overlapping time windows of past observations, which served as input sequences for predicting future values.

### 2.2. Analysis of Mutual Information and Correlation

Understanding the relationships between meteorological and geodetic variables is crucial when developing accurate precipitation prediction models. In particular, when working with GNSS-derived datasets, such as ZPD, ZHD, ZWD, and PWV, it is essential to assess their influence on observed precipitation values.

To assess these relationships, both Pearson correlation and Mutual Information (MI) were employed. Correlation measures linear dependencies between variables; however, it may fail to detect nonlinear interactions that are often present in atmospheric dynamics. Mutual information, on the other hand, provides a more general framework by quantifying the amount of information shared between two variables, regardless of the linearity of their relationship.

**Figure 1:**
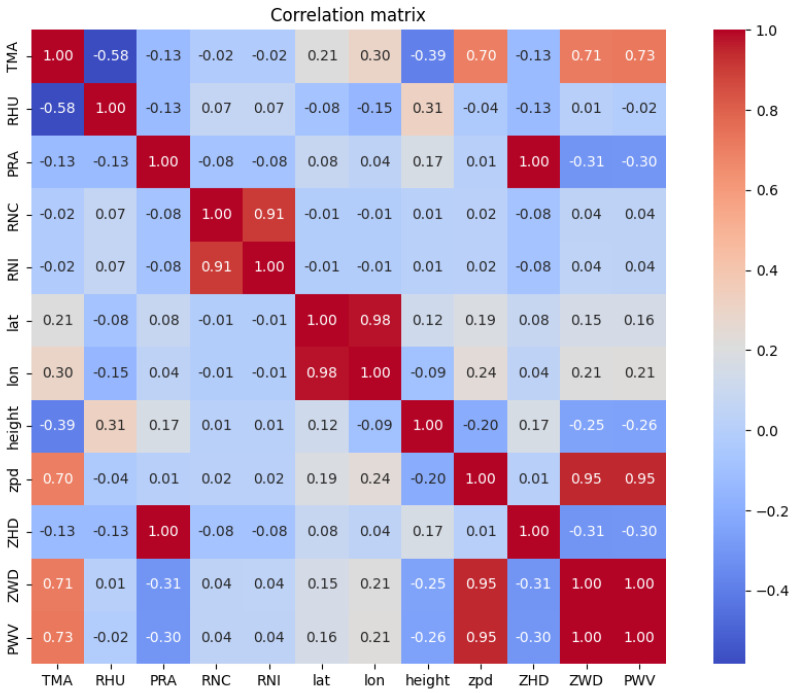
Correlation between observed parameters referring to the same time.

Analysis of the correlation matrix (Figure 1) clearly indicates that there is no significant linear correlation between precipitation measurements and the other atmospheric variables, including their lagged versions.

**Figure 2:**
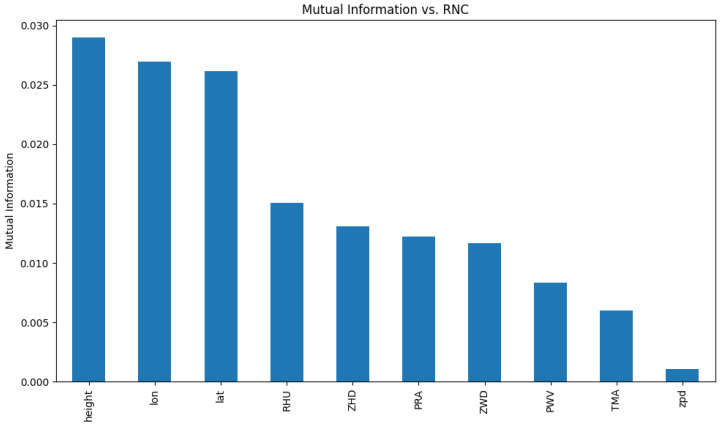
Mutual information of variables vs. precipitation.

To capture more complex dependencies, mutual information regression was applied using an opportune function from the scikit-learn library [24]. This function is based on the method proposed in [25], which estimates mutual information through entropy calculations derived from k-nearest neighbors. This approach allows for the investigation of statistical dependencies, if any, including nonlinear relationships, between the predictor variables and the target variable (RNC). Please refer to Figure 2 for a view of the mutual information between the available variables and precipitation.

**Figure 3:**
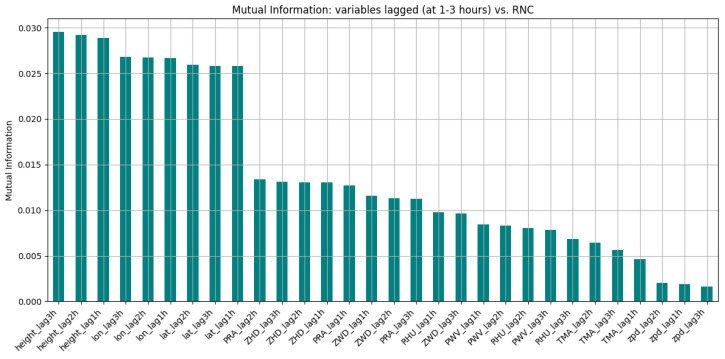
Mutual information of variables vs. precipitation including lagged values.

Given the dataset’s temporal resolution (one measurement every 30 s), lagged features were generated at 1 h, 2 h, and 3 h intervals (corresponding to 120, 240, and 360 time steps, respectively). These lagged variables serve to incorporate temporal memory into the model, which is essential when employing sequence-based neural networks such as Long Short-Term Memory (LSTM) models. The mutual information between precipitation at time *t* and the lagged variables has also been computed, as shown in Figure 3.

Interestingly, mutual information values were relatively high for variables such as latitude, longitude, and geodetic height parameters, which are recalculated at each epoch by GNSS processing software. The fluctuations of these parameters, although not directly related to precipitation, may reflect changes in atmospheric water vapor and other meteorological and non-meteorological factors captured indirectly by the GNSS signal delay.

In conclusion, neither linear correlation nor mutual information show relevant features in predicting precipitation, suggesting that direct application in regression-based machine learning approaches would not yield significant improvements.

In time series analysis, there are two main approaches to using past data to predict future values: lagged variables and sequence models like LSTM (Long Short-Term Memory). While both methods rely on historical data, they treat time very differently.

Lagged variables are simply past values of a variable shifted backward in time. These lagged values are treated as independent input features by machine learning models. This technique is suitable for traditional models like Linear regression, Random Forest, and XGBoost. However, these models do not understand time as a sequence—they just see the lagged features as separate columns without any inherent time ordering or continuity.

LSTM is a type of Recurrent Neural Network (RNN) that is specifically designed to handle sequences of data. Instead of flattening time into static lagged columns, you provide the model with a continuous time window of past observations. The LSTM processes each time step in order, learning temporal patterns, such as trends, sudden changes, and long-term dependencies. This allows the model to make more informed predictions because it understands how the variables evolve over time.

### 2.3. Experiments with LSTM

Before training an LSTM, time series data needs to be formatted into sequences that the model can process. To achieve this, the dataset has been structured into overlapping windows of past observations, which serve as the input for predicting future values, to transform raw data into sequences with a defined lookback window and forecast horizon. LSTM require 3D input (shaped as samples, timesteps, and features), but typical datasets are in a 2D format (samples, features). It is also possible to convert time series data into sliding windows of past observations to predict future values using the following functions:

- Lookback window (lookback): Defines how many past time steps to consider as input.
- Forecast horizon (delay): Determines how far ahead we want to predict.
- Step size (step): Controls how frequently sequences are sampled.

The function iterates over the dataset, extracting overlapping windows of lookback past observations, and assigning a target value (y) from delay future steps. In this case, the target is computed as the maximum value within the forecast horizon, allowing the model to capture future peaks.

- Input (X): Each sequence consists of historical lookback time steps, allowing the model to recognize trends and temporal patterns. In the case study, data from the previous eight hours were taken.
- Output (y): Instead of predicting the next immediate value, the target is the maximum value in the next delay steps, which allows the model to anticipate future peaks.

By structuring the data in this way, LSTMs can take advantage of past dependencies to make accurate forecasts, capturing both short-term fluctuations and long-term trends in the time series.

After a severe trial, the model LSTM used has two layers, meaning it passes sequences to the next layer instead of just the final hidden state and it includes a Dropout layer, which helps prevent overfitting by randomly deactivating some neurons during training.

## 3. Results

### 3.1. Performances of LSTM

In the first experiments, the input variables considered were TMA, RHU, PRA, PWV, and RNC, along with time-related features such as month and hour. The target variable was the RNI value one hour after the prediction time.

**Figure 4:**
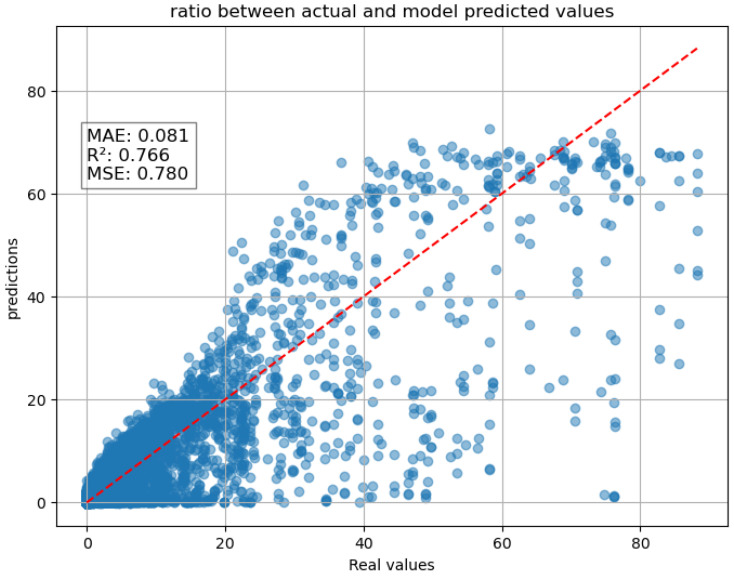
Scatter plot one-hour forecast of the LSTM with the RNC as input.

The scatter plot below (Figure 4) compares real values and values predicted by the model.

The plot shows a concentration of points near lower values, meaning the model performs well for smaller rainfall intensities but struggles with higher intensity values. The dispersion increases as real values increase, indicating that the model underestimates high-intensity rainfall (many predicted values are lower than expected). However, if we consider the model metrics, we have a very low mean absolute error (mae) of 0.081, suggesting that, on average, predictions are relatively close to actual values. A mean square error with a value of 0.78 suggests that while the model does make errors, they are not excessively large. However, given that rainfall intensity can vary significantly, the presence of outliers (as seen in the scatter plot) may contribute to this value. The model performs well overall, capturing a significant portion of the variance in rainfall intensity (R^2^ = 0.762). It tends to underestimate extreme rainfall events, which is common in weather prediction models due to the complexity of atmospheric dynamics. A key observation on the model’s performance is its strong dependence on the cumulative precipitation variable (RNC). In fact, RNC and RNI are highly correlated. Because RNC is cumulative by definition, it directly encodes past precipitation and thus becomes highly predictive in sequence-based models. In other words, correlation analysis at a single time step underestimates the predictive role of RNC, whereas in time series forecasting, its cumulative structure makes it a dominant predictor.

**Figure 5:**
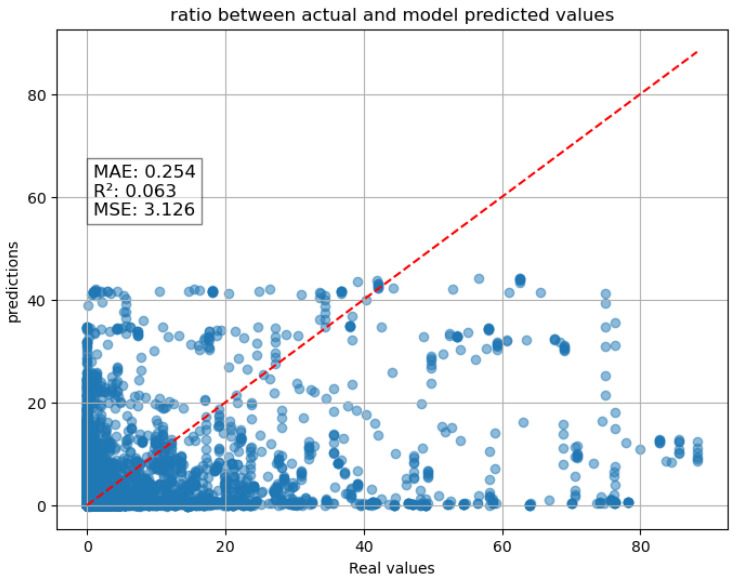
Scatter plot one-hour forecast of the LSTM without the RNC as input.

When this feature is removed from the input data, the model’s predictive performance drastically worsens. This suggests that the model attaches considerable importance to this variable over the others, relying heavily on it for accurate predictions.To illustrate this effect, the scatter plot below (Figure 5) shows the performance of the model without RNC as an input variable, clearly showing a strong degradation in predictive accuracy when this feature is excluded.

### 3.2. Performances of RFR

It is important to say that cumulative precipitation cannot always be directly measured at the location of the GNSS receiver. This is because there may not be a rain gauge exactly at that position. In contrast, variables such as pressure, temperature, and humidity can be estimated more reliably by interpolating data from nearby weather stations.

An interesting result emerged from the use of an RFR model for instantaneous precipitation intensity estimation at the same time step. Although it lacks the temporal modeling capabilities inherent to recurrent neural networks, the RFR proved effective in identifying the most influential input variables driving rainfall estimations. Its strength lies in capturing complex, nonlinear relationships between features, offering valuable insights into the factors influencing precipitation. The model was trained using 40 decision trees with a maximum depth of 20.

**Figure 6:**
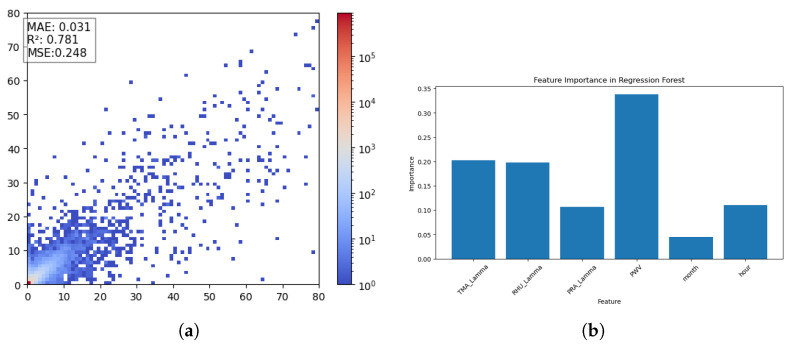
(**a**) Hexbin plot of instantaneous prediction of RNI. (**b**) Graph of the importance of each feature for RFR.

The graph on the left (Figure 6) is a Hexbin scatter plot, where instead of plotting individual points, group data are plotted into hexagonal bins and are colored based on the number of points inside each bin. Instead, the graph on the right illustrates the score for all input features, highlighting the extent to which each one has an effect on the model’s ability to predict the rainfall.

Although the model performs well, it is important to note that predictions on training data yield significantly better results, with an MAE test of 0.018, MSE test of 0.055, and R^2^ test of 0.949. This suggests that the model does not fully generalize to new data and may struggle with unseen cases. To further assess this, we reduced the tree depth to max depth, equal to 15, which resulted in a more balanced performance. With this adjustment, the test set achieved an R^2^ of 0.67, MAE test of 0.049, and MSE test of 0.375, while the training set reported an R^2^ of 0.83, MAE train of 0.040, and MSE train of 0.185. Table 2 reports the performance of RF models train with different maximum three depth (the difference in depth does not make a significant difference to the final model, which we will see later).

From the results obtained, what catches the eye is how the model considers PWV as one of the key factors in rainfall estimation, highlighting its importance in the forecasting process and consequently in GNSS measurements.

### 3.3. The Two-Step Machine Learning Approach

**Figure 7:**
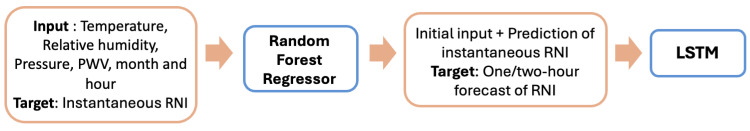
Scheme of two-tier system of RFR + LSTM.

In the light of this, the approach was to develop a two-tier system that integrates a Random Forest model with the previously discussed LSTM model (Figure 7). In this setup, the Random Forest model processes the input information provided by the weather station (excluding rainfall) and GNSS receiver observation performs an instantaneous RNI estimation at the same time step, used as a preprocessing step to reduce data noise. This estimation is then incorporated as an additional input for the LSTM model.

**Figure 8:**
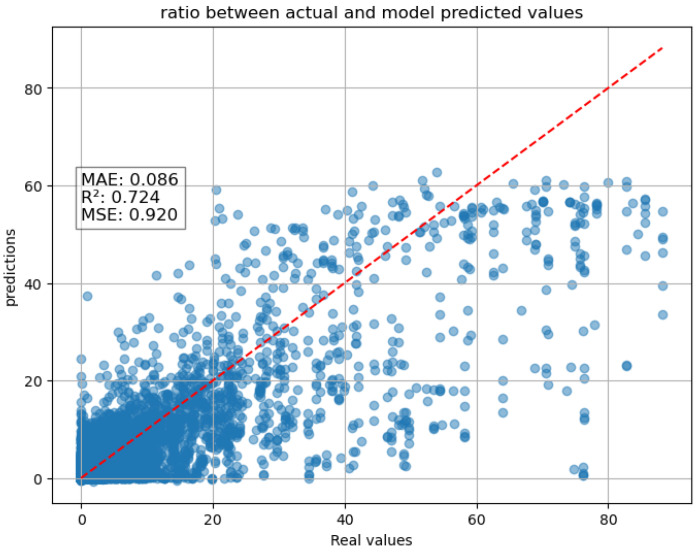
Scatter plot of two-tier system of RFR + LSTM one-hour forecast.

The result obtained is illustrated in the graph displayed below (Figure 8). This scatter plot can be directly compared with the one obtained using the LSTM model without any rainfall information as input (Figure 5). From the analysis of this comparison, a significant improvement in performance clearly emerges. This suggests that the integration of the RFR has provided a crucial contribution, enhancing the overall accuracy of the model and its generalization capability for the test data.

**Figure 9:**
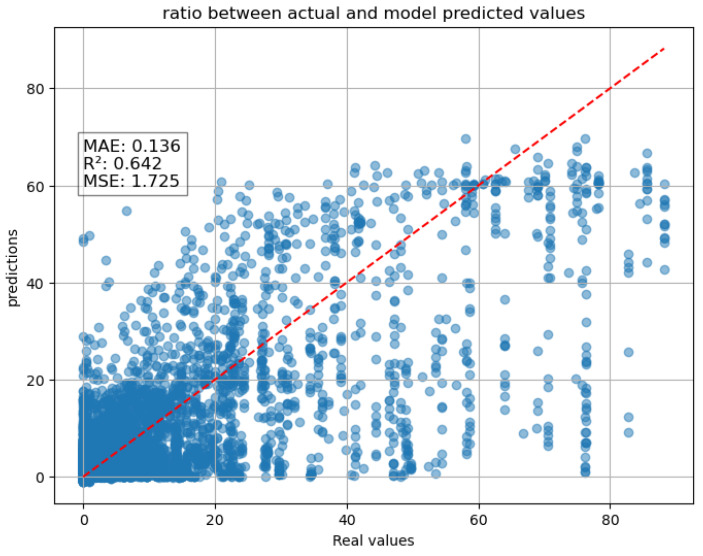
Scatter plot of two-tier system of RFR + LSTM two-hour forecast.

A 2-h forecast of maximum rainfall was also tested, using input data from the previous 12 h (lookback). Although the forecast accuracy decreases compared to other scenarios, we can see from the graph below a greater dispersion; however, the results remain satisfactory, with an R^2^ of 0.64 and an MAE of 0.13 (Figure 9). The performance of the models was also influenced by the imbalance between rainy and dry days, which likely contributed to reduced accuracy in detecting precipitation events, particularly the most intense ones.

As we can see from the graphs and results, the model provides reasonably good estimates but remains imprecise, particularly for higher rainfall values, indicating difficulties in accurately predicting extreme events. However, given the inherent challenges of precipitation forecasting due to its high variability and the influence of multiple atmospheric factors, the results obtained are hopeful, especially when compared to instruments used today such as radar [26].

**Figure 10:**
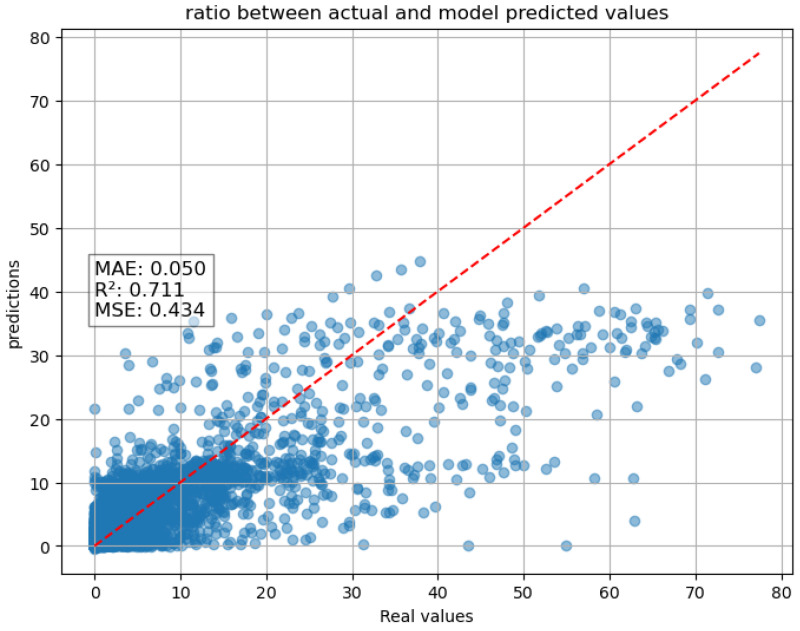
Scatter plot of two-tier system of RFR + LSTM one-hour forecast of average RNC.

Another experiment was conducted by changing the target variable of the model, shifting the focus to predicting the average cumulative precipitation instead of the maximum instantaneous rainfall intensity (Figure 10). Estimates of cumulative rainfall give a broader perspective on total rainfall accumulation over a given period. This adjustment allowed for a broader understanding of total rainfall accumulation, which is also particularly useful for applications such as water resource management.

**Figure 11:**
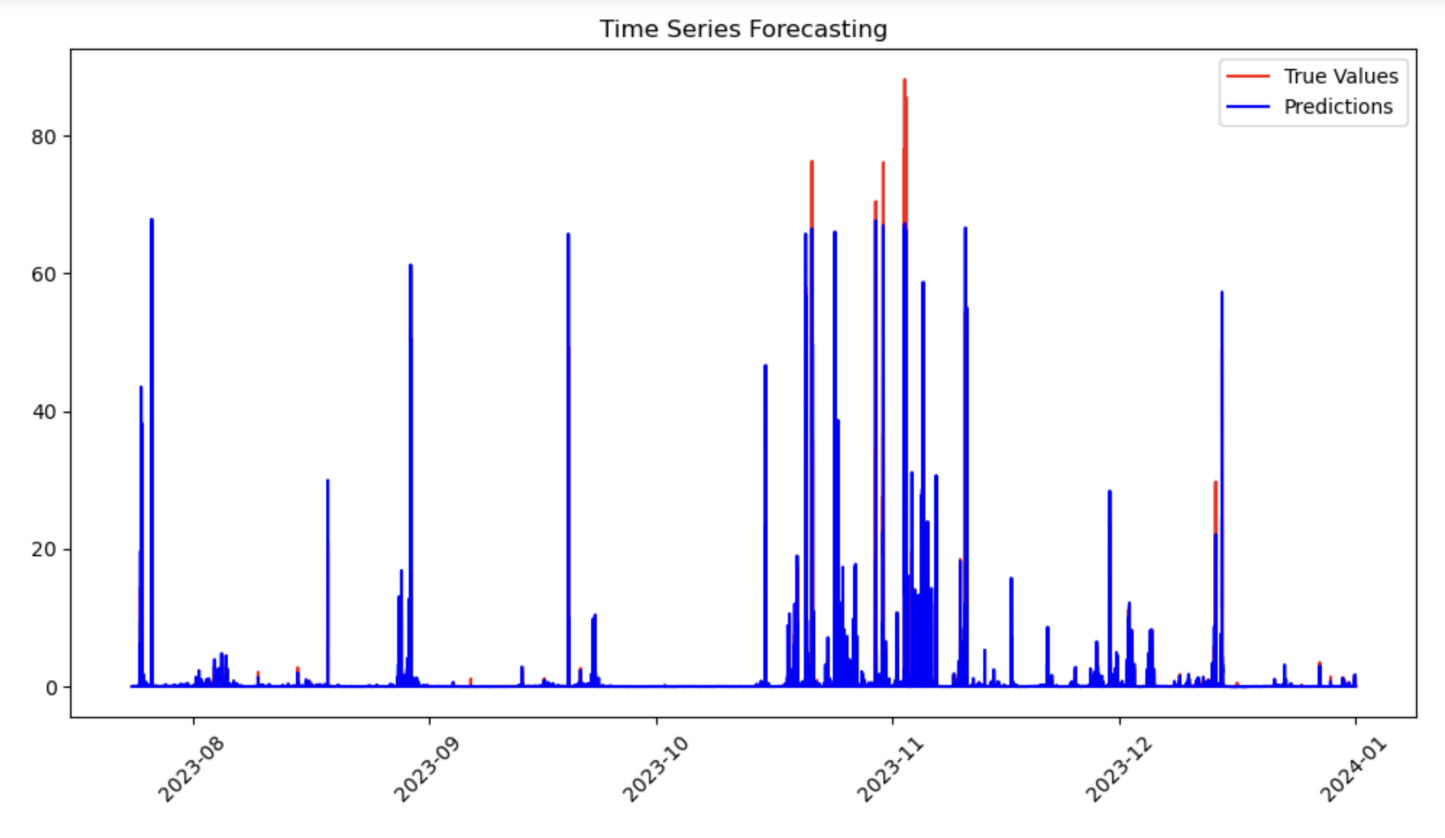
One-hour forecast comparison with RFR + LSTM data (test data).

The results obtained from this modified approach were very similar to those achieved when forecasting the maximum value of RNI. This suggests that the model is capable of producing good estimates for predicting RNC values, reinforcing its generalization ability among different rainfall prediction tasks. To get a better picture of the result, it is possible to see a comparison between the predicted and real values of the series (Figure 11).

Overall, the proposed model demonstrated good predictive performance, but with some limitations. As previously observed, some predictions were underestimated, indicating that the model struggles to forecast more intense events. This is a common issue in predictive models, as extreme events are rare in the training data, and the models tend to be more conservative when dealing with phenomena that occur infrequently. This highlights the need for more sophisticated approaches to handle low-probability yet high-impact situations, such as extreme weather events.

## 4. Discussion

While the use of an LSTM neural network yielded promising results in modeling and predicting precipitation dynamics, a strong dependence on the availability of the RNC variable (cumulative rainfall) was observed. Specifically, the model’s performance degraded significantly when this variable was removed from the feature set. This finding suggests that the RNC variable carries substantial predictive power, potentially encoding critical information about short-term precipitation dynamics that other variables, whether atmospheric or derived from GNSS, do not fully capture.

In this context, the RFR emerged as a valuable tool during the preprocessing phase. The RFR model was able to provide meaningful estimates even in the absence of RNC, thereby offering a viable strategy for imputing or approximating the missing signal. Furthermore, the ensemble nature of the Random Forest algorithm allows it to capture complex, nonlinear interactions among features and perform implicit feature selection, which can help identify the most relevant variables and filter out noisy or redundant information.

An important insight from this study is that the RFR model, despite not being ideally suited for sequential data, can still enhance the performance of deep learning models like LSTM when used strategically. By training the RFR on the available features and using its predictions as additional inputs, or as a replacement for missing RNC data, the input space to the LSTM becomes cleaner and more structured. This approach can mitigate overfitting, reduce the influence of irrelevant fluctuations, and guide the LSTM toward learning more generalizable temporal patterns.

Moreover, the predictive outputs of the RFR model may offer interpretability benefits. By examining feature importances or partial dependence plots, it becomes possible to extract insights into which variables or lagged features are contributing most to the prediction of precipitation events. This information can inform future sensor deployment strategies or highlight the value of specific GNSS-derived variables such as ZWD, ZHD, or PWV.

Another consideration is the complementary strengths of both models. While LSTM networks excel in capturing temporal dependencies and long-term trends, Random Forests are robust to noisy data and perform well with a limited amount of training data. Therefore, a hybrid approach combining Random Forests for noise reduction and feature refinement with LSTM for temporal sequence modeling could offer a robust framework for forecasting extreme precipitation events, particularly in scenarios where key variables may be missing, unreliable, or delayed in real-time systems.

Finally, future studies could explore ensemble architectures that dynamically integrate the outputs of both models, or adopt model stacking strategies to optimize prediction performance under varying data availability conditions. In operational settings, such adaptability could be particularly valuable for early warning systems or meteorological forecasting in data-constrained environments.

## 5. Conclusions

This study proposes a two-step system for predicting the maximum rainfall intensity and cumulative rainfall in the next two hours. Input variables include temperature, relative humidity, and pressure, collected from a surface weather station, as well as PWV, calculated indirectly processing data from a co-located GNSS scientific receiver. In particular, an RFR model is used to provide an instantaneous RNI, which successfully captures the dependence between PWV and RNI. The output of this model is then added as input, along with the other variables, to an LSTM model. This method reduces the noise between the data, allowing for improved prediction in the absence of the RNC input to the LSTM model. We also noticed that the RFR needs a lot of data to work; with little data available, it cannot make a good prediction. Although the results are not yet optimal in some cases, as the model remains conservative with the difficulty of predicting extreme events that are not very frequent (fortunately), this study nevertheless provides promising insights. In particular, given the uncertainty surrounding the short-term correlation between PWV and RNI, this study demonstrates how a supervised model like RFR can effectively capture this dependency. In fact, although no direct correlations or mutual information were found between the selected parameters and future precipitation—thus limiting their direct application in regression-based machine learning approaches—the use of LSTM techniques enables their effective exploitation, as demonstrated in this study. It is important to have a tool that can very accurately predict precipitation in the next one or two hours, especially in relation to critical situation and alerts, thus enabling the timely activation of emergency measure, especially for risk management authorities (e.g., for the organization of intervention teams) as well as timely warning of the population.

Machine learning techniques can be a powerful tool for improving the accuracy and timeliness of predictions. These models are faster and less computationally expensive than traditional methods. However, they still lack the reliability of physical models and are not yet capable of generalizing well enough to predict rare events. As a result, physical simulations remain essential. Their importance becomes even more evident in the face of the worsening of climate change.

The adoption of a two-step strategy, combining RFR and LSTM, proved effective in addressing the lack of local precipitation observations by reconstructing RNC at time t and subsequently exploiting temporal dependencies for improved nowcasting. For future research, it would be useful to explore different hybrid model configurations that can optimally combine the strengths of both approaches. In addition, the incorporation of additional historical data, by extending the observational period and introducing spatial data (such as ERA5 [27]) or additional meteorological variables, as well as feature-engineering techniques, such as the introduction of the gradient taking into account the variability (both temporal and spatial) of PWV, would make it possible to identify whether the amount of PWV is increasing or decreasing in the time, an indication of atmospheric change. These solutions could further refine the model and improve its generalization capability, and would significantly enhance the model’s ability to generalize and improve its performance in forecasting high-intensity precipitation events. Addressing the imbalance between rainy and dry days through resampling strategies, cost-sensitive learning, or the extension of the dataset will be a key step toward improving the robustness and predictive accuracy of future models, particularly for intense precipitation events. The full code used can be found on Github at the following link: https://github.com/Lolop97/Prediction-rainfall (accessed on 15 April 2025).

## Figures and Tables

**Table 1:** Summary of the main settings of the MG-APP software used for ZPD retrieval.

Setting Category	Adopted Option
Filter Type	Kalman filter and Square Root Information Filter (SRIF)
Tropospheric Model	Saastamoinen
Mapping function	VMF1 (Vienna Mapping Function 1)
Ocean tide model	FES2004
Constellations	GPS, GLONASS, BeiDou and Galileo
Processing Mode	Postprocessed (forward–backward filtering)
Processing scheme	Precise Point Positioning (PPP) via precise products
Pseudorange Handling	Phase-smoothed pseudorange enabled to improve accuracy
Data Handling	Auto-recognizes RINEX versions (2.x and 3.x)
Downloaded required products	Final multi-GNSS orbit and clock solution (COM) produced by CODE (Center for Orbit Determination in Europe)

**Table 2:** Performance of Random Forest models with different maximum depths.

Max_DEPTH RF	R^2^ Test	MAE Test [mm/h]	MSE Test [mm^2^/h^2^]	R^2^ Train	MAE Train [mm/h]	MSE Train [mm^2^/h^2^]
17	0.73	0.04	0.31	0.90	0.03	0.10
16	0.70	0.04	0.33	0.87	0.03	0.14
15	0.67	0.05	0.37	0.82	0.04	0.18
14	0.62	0.05	0.42	0.77	0.05	0.25
13	0.57	0.06	0.48	0.70	0.05	0.32

## Data Availability

The raw data supporting the conclusions of this article will be made available by the authors on request.
